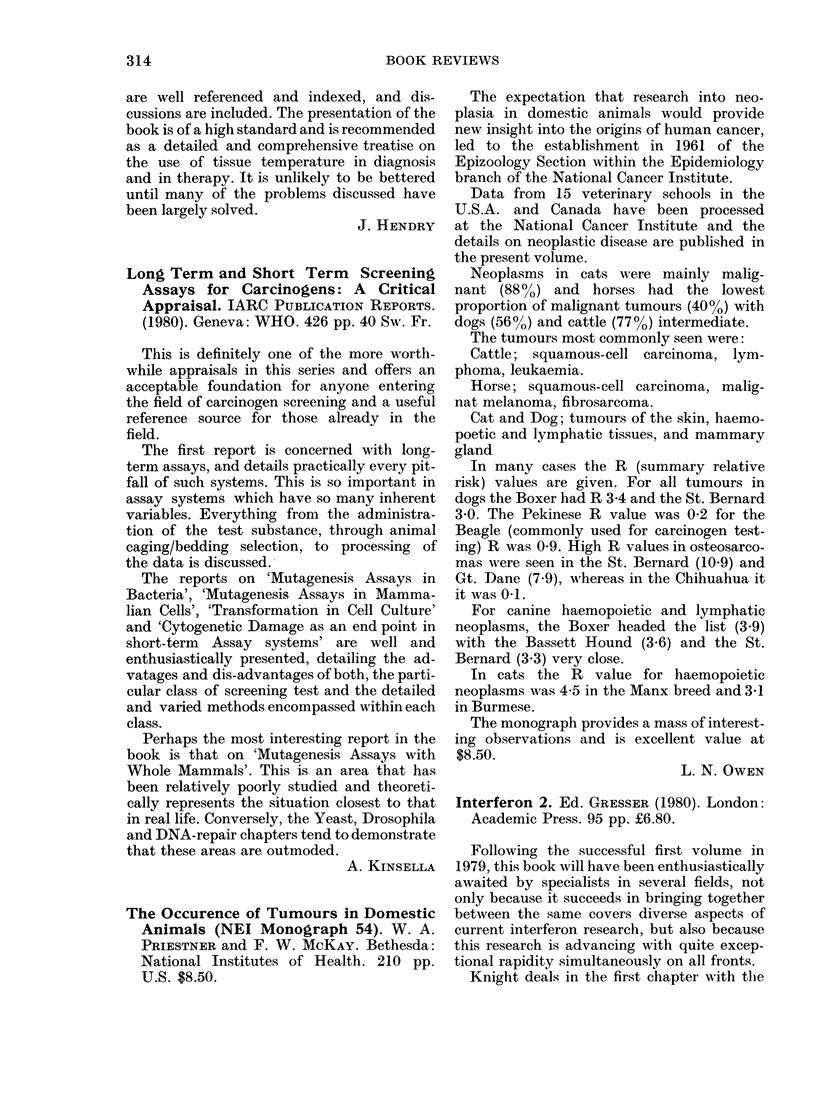# The Occurence of Tumours in Domestic Animals (NEI Monograph 54)

**Published:** 1981-08

**Authors:** L. N. Owen


**The Occurence of Tumours in Domestic Animals (NEI Monograph 54).** W. A. PRIESTNER and F. W. MCKAY. Bethesda: National Institutes of Health. 210 pp. U.S. $8.50.

The expectation that research into neoplasia in domestic animals would provide new insight into the origins of human cancer, led to the establishment in 1961 of the Epizoology Section within the Epidemiology branch of the National Cancer Institute.

Data from 15 veterinary schools in the U.S.A. and Canada have been processed at the National Cancer Institute and the details on neoplastic disease are published in the present volume.

Neoplasms in cats were mainly malignant (88%) and horses had the lowest proportion of malignant tumours (40%) with dogs (56%) and cattle (77%) intermediate.

The tumours most commonly seen were:

Cattle; squamous-cell carcinoma, lymphoma, leukaemia.

Horse; squamous-cell carcinoma, malignat melanoma, fibrosarcoma.

Cat and Dog; tumours of the skin, haemopoetic and lymphatic tissues, and mammary gland

In many cases the R (summary relative risk) values are given. For all tumours in dogs the Boxer had R 3·4 and the St. Bernard 3·0. The Pekinese R value was 0·2 for the Beagle (commonly used for carcinogen testing) R was 0·9. High R values in osteosarcomas were seen in the St. Bernard (10·9) and Gt. Dane (7·9), whereas in the Chihuahua it it was 0·1.

For canine haemopoietic and lymphatic neoplasms, the Boxer headed the list (3·9) with the Bassett Hound (3·6) and the St. Bernard (3·3) very close.

In cats the R value for haemopoietic neoplasms was 4·5 in the Manx breed and 3·1 in Burmese.

The monograph provides a mass of interesting observations and is excellent value at $8.50.

L. N. OWEN